# Strengthening community-clinical linkages to reduce cardiovascular disease risk in rural NC: feasibility phase of the CHANGE study

**DOI:** 10.1186/s12889-020-8223-x

**Published:** 2020-02-21

**Authors:** Carmen D. Samuel-Hodge, Ziya Gizlice, Sallie D. Allgood, Audrina J. Bunton, Amber Erskine, Jennifer Leeman, Samuel Cykert

**Affiliations:** 1 Gillings School of Global Public Health, Department of Nutrition, Center for Promotion and Disease Prevention, University of North Carolina at Chapel Hill, 1700 Martin Luther King Jr. Blvd., Room 216, CB #7426, Chapel Hill, NC 27599-7426 USA; 2 Center for Health Promotion & Disease Prevention, 1700 Martin Luther King Jr. Blvd. CB# 7426, Chapel Hill, NC 27599-7426 USA; 3 School of Nursing, University of North Carolina at Chapel Hill, Carrington Hall, CB # 7460, Chapel Hill, NC 27599-7460 USA; 4 Cecil G Sheps Center for Health Services Research, 725 Martin Luther King Jr. Blvd. CB# 7590, Chapel Hill, NC 27599-7590 USA; 5 School of Medicine, Division of General Medicine and Clinical Epidemiology, University of North Carolina at Chapel Hill, 145 N Medical Drive, CB# 7165, Chapel Hill, NC 27599-7165 USA

**Keywords:** Rural health services, Lay health advisors, Social determinants of health, Prevention and control

## Abstract

**Background:**

Community Health Workers (CHW) are recommended for delivery of interventions to prevent cardiovascular disease, but there is insufficient evidence to guide implementation of CHW interventions in rural, medically underserved areas.

**Methods:**

Using a hybrid implementation-effectiveness design, we evaluated the implementation and effectiveness of an adapted, evidence-based cardiovascular disease risk reduction intervention among rural high-risk adults. CHWs at a community health center and local health department recruited, enrolled and counseled participants during 4 monthly home visits and 3 brief phone contacts. Participant data collection included pre- and post-intervention measurements of blood pressure, weight, and dietary and physical activity behaviors. We evaluated implementation with measures of intervention reach and delivery fidelity. Statistical analyses included descriptive statistics and paired t-tests.

**Results:**

Study participants (*n* = 105) had a mean age of 62 years and included 88% Non-Hispanic Blacks and 82% females. Recruitment strategies resulted in the enrollment of 38% of interested and eligible participants who received 80% of the planned intervention visits and phone contacts. Mean differences in pre−/post-intervention measures showed significant mean reductions in blood pressure (− 5.4 mmHg systolic, *p* = .006; − 2.3 mmHg diastolic, *p* = .04) and body weight (− 3.8 lb., *p* = .02). Self-reported dietary and physical activity behaviors also improved significantly.

**Conclusion:**

This feasibility study demonstrated preliminary implementation and program effectiveness of a CHW-delivered intervention to reduce cardiovascular disease risk factors. Additionally, it identified areas for future refinements to strategies that strengthen community-clinical linkages with an integrated role of CHWs in rural health care delivery. If results from this feasibility study can be enhanced in a larger sample, there would be significant potential to positively impact the excess burden of chronic diseases that adversely impact rural, low-income, and medically underserved populations.

**Trial registration:**

ClinicalTrials.gov: NCT03582696.

## Background

The leading cause of death in the US is cardiovascular disease (CVD), with the greatest CVD burden concentrated in the southeastern states [1–3]. Within this geographic region, CVD rates are highest among African Americans, [4] Native Americans, those with lower socioeconomic status, [5] and those living in rural communities [6]. Factors that contribute to high CVD rates in these populations include consuming fewer fruits and vegetables, [7, 8] engaging in less leisure-time physical activity, [9] and having more limited access to healthcare [10, 11] compared to their higher income, non-minority, urban/suburban, and non-southeastern counterparts.

About 40% of North Carolinians or approximately 4 million people live in one of the state’s 80 rural counties [12]. Despite increased risk for CVD in rural Americans, few CVD prevention interventions are available for rural populations [13–15]. To address this gap, we developed and tested the Carolina Heart Alliance Networking for Greater Equity (CHANGE) intervention, which combines an evidence-based behavior change counseling intervention with strategies to link clinical and community services to prevent CVD in a rural county in the Southeastern US. The CHANGE intervention is designed to be delivered by Community Health Workers (CHWs), defined as frontline public health workers who are trusted members of and/or have an unusually close understanding of the community served [16]. Prior research has demonstrated the effectiveness of CHW-led interventions at reducing cardiovascular disease risk factors, [15]. However, little is known about the feasibility of implementing CHW-delivered interventions in rural settings, especially among rural minority populations. While there is some CVD risk reduction intervention research in rural settings, [17–19] evidence gaps remain for studies with CHWs among rural African Americans. Moreover, in clinical-community linkages research with CHWs, [20] more evidence is needed on the effectiveness of CHWs working “interchangeably in both community and healthcare settings” to determine whether they can build and enhance these linkages [15]. This feasibility study aims to address these evidence gaps with preliminary data.

This report describes the feasibility phase of a hybrid implementation-effectiveness study [21] designed to test the implementation and effectiveness of the CHANGE intervention in one predominantly African American, rural county’s community health center and health department. With this type of hybrid implementation-effectiveness study design, the primary aim is to test the effectiveness of implementation strategies, while the intervention’s impact on relevant clinical outcomes is secondary [21]. The purpose of the feasibility phase was to assess the CHANGE intervention’s implementation and effectiveness outcomes with the goal of refining implementation protocols prior to testing the CHANGE intervention in a larger sample of predominantly African American, rural adults.

## Methods

Using a hybrid implementation-effectiveness design for this feasibility study, we evaluated the implementation and effectiveness of the CHANGE program with a single arm, pre−/post-study design, [22] where participants were measured before and immediately after receiving the intervention. The University of North Carolina (UNC) Non-Biomedical Institutional Review Board (IRB) approved and monitored the study, beginning with approval in January 2016; direct interaction with study participants ended in September 2017. All participants provided written informed consent, and clinic patients consented to have study staff obtain CVD-related lab values from their medical record by signing a separate Health Insurance Portability and Accountability Act (HIPAA) consent form. For participants enrolled at the health department, no information was gathered from their medical record.

### The CHANGE intervention

Two CHWs delivered an adapted version of the evidence-based Heart-to-Health lifestyle intervention [23] and referred participants to community and clinical resources. Heart-to-Health is a low-intensity behavioral lifestyle intervention targeting CVD risk reduction through dietary and physical activity behavior changes, smoking cessation, and medication adherence. To support these behavioral changes, CHANGE also included a community ‘heart healthy’ resource guide and protocols for referring participants to and following up on their use of those resources. CHWs delivered the CHANGE intervention over 4 monthly, in-person counseling visits (45–60 min) in participants’ homes or at local venues selected by the participant. Between these monthly counseling visits, the CHW made short ‘booster calls’ (about 15–20 min) to follow up with participants on the progress made with goals set and actions taken on referrals made at the last counseling visit. Each participant received a program manual with educational materials on healthy eating, taking medicine, physical activity, stopping smoking, and a community resources directory including resources in their community related to heart health, health care, and transportation services. To maximize the potential benefits of lifestyle changes, program topics were introduced to participants based first on the participant’s selection of the behavior they most wanted to change, then on the potential CVD risk reduction expected by making the behavior change (ranked from highest to lowest). See Table 1 for more details on the CHANGE program content (4 main areas), listed in order of importance for CVD risk reduction. The total planned contact time (4 counseling visits + 3 booster calls) for this low-intensity intervention is estimated at 4 to 6 h.

**Table 1:** CHANGE Program Content and Contacts^a^

Program Contacts	Program Content^b^
Counseling Visit 1 [45–60 min duration]	▪ Written informed consent (HIPAA consent if clinic patient)
	▪ Baseline study measurements (weight, blood pressure, survey)
	▪ CVD Risk Score calculation
	▪ **Taking Medications Module**
	• What you should know
	• Reasons for not taking medication and ways to address
	• Local pharmacies
	▪ Goal-setting and action planning
	▪ Referrals to community resources (as needed)
Counseling Visit 2 [45–60 min duration]	▪ **Stopping Smoking Module**
	• What Works (QuitlineNC, Asking for Support, Medicines)
	▪ Goal-setting and action planning
	▪ Referrals to community resources (as needed)
Counseling Visit 3 [45–60 min duration]	▪ **Healthy Eating Module**
	• Nuts, Oils, Dressings and Spreads
	• Vegetables, Fruits, Beans and Whole Grains
	• Drinks, Desserts, Snacks, and Eating Out
	• Fish, Meat, Dairy and Eggs
	▪ Goal-setting and action planning
	▪ Referrals to community resources (as needed)
Counseling Visit 4 [45–60 min duration]	▪ **Physical Activity Module**
	• Walking
	• Keep Walking and Moving More
	• Stay on Track
	• Add Muscle Strengthening
	▪ Follow-up measurements and survey administration
Booster Calls 1–3 [15–20 min duration]	▪ Check-in on goal progress (successes and challenges related to the topic(s) covered)
	▪ Check-in on referrals (actions taken or barriers to following through)
	▪ Reminder for next counseling session

^a^Monthly counseling visits were designed as home visits or in-person visits at a community location selected by the participant. Booster calls were scheduled for 10–14 days after each of the first 3 counseling visits

^b^Content modules are ordered by highest to lowest potential to reduce CVD risk. No more than 2 topics were covered at each counseling visit

### Site, CHW, and participant recruitment

The two sites selected for our feasibility study included a Federally Qualified Health Center (FQHC) and a local health department in Hertford, a rural NC county. We selected Hertford county because of its high rates of CVD risk factors and our prior relationships with FQHC leadership. Hertford county is located in the northeastern region of NC, with a population of about 24,000, poverty rate of 26%, and over 60% of the population self-identified as African American [24]. In 2016, Hertford County was ranked 89th for health outcomes and 93rd for health behaviors, among NC’s 100 counties [25]. The research team created subcontracts with both sites to cover the costs of staff member participation on the community-engaged research team and the salary and benefits for a full-time CHW.

The CHANGE Study’s enrollment goal for Hertford County was 150 participants. This sample size would provide a reasonable estimate of feasibility as measured by recruitment and attrition rates, while allowing for a loss to follow-up of 20%. To be eligible, participants had to: live in or receive medical care in Hertford County, North Carolina; be 18–80 years of age; and speak English. Women who reported that they were pregnant were excluded or withdrawn, as pregnancy may account for observed changes in weight and blood pressure. The CHW at the health department recruited participants through community outreach, including strategies such as word-of-mouth, flyers, local newspaper or magazine advertisements, participation in health fairs and community events, and visits to churches, beauty salons, and senior centers. Recruitment by the health department was tilted toward primary prevention of CVD which meant anyone encountered who was interested and eligible to participate was invited. In contrast, the CHW at the FQHC recruited through the electronic health record systems, with a focus on secondary prevention. Clinic nurses pre-screened existing patients for elevated risk of a cardiac event, and then created a list that the CHW used to recruit study participants, either at a clinic visit or via a phone call. Patients were eligible to participate if they were smokers or had uncontrolled diabetes (A1c greater than 8%), hypercholesterolemia (low density lipoprotein [LDL] greater than 130 mg/dL), hypertension (systolic blood pressure > 140 or diastolic > 90 mmHg) or a previous cardiovascular event. Patients who appeared in multiple risk categories were prioritized.

### Staff training for intervention delivery

Prior to patient recruitment, the research team conducted an intensive 6-day centralized study training with the staff responsible for participant recruitment and intervention delivery (site supervisors and CHWs). The training sessions included reviews of study protocols, informed consent and participant confidentiality, participant recruitment and study site protocols, CHANGE intervention content, community referral resources, and data collection methods. Training also included opportunities for CHWs to practice motivational interviewing skills and to role-play enrolling, counseling, and referring study participants.

### Data collection

Data collection included measures of both implementation and effectiveness outcomes; methods for each type are detailed below. The CHWs collected all participant data at counseling visits. For health center patients, eligibility screening data used to rank patients by CVD risk factors was provided to the study staff through a data sharing agreement.

#### Implementation outcomes

Data were collected to assess reach and delivery fidelity. Data on reach were captured thorough tracking logs that CHWs maintained of the number of individuals they invited to participate, whether they agreed to participate, and reasons for declining. Data on fidelity were collected through an online system where CHWs documented delivery of the intervention including contact duration, content covered, goals set, referrals made to community resources, and disposition of referrals given (action taken and/or services received).

#### Effectiveness outcomes

CHWs collected blood pressure, weight, self-reported dietary and physical activity data to measure program effectiveness. They collected outcome measures at the first and last intervention visits (Home visits 1 and 4). With this study’s primary focus on the effective implementation of the CHANGE program, and a secondary focus on its effectiveness in reducing CVD risk, we intentionally limited our data collection to reduce the burden on both the CHW and participants. Data collection included physical measures of weight and blood pressure, brief validated surveys of dietary and physical activity behaviors, and general demographic and health information. Weight, as the average of two measures, was assessed in pounds to the nearest tenth, by an electronic scale (Seca 874, Seca, Hanover, MD). Blood pressure (BP) measurements were taken with an automated BP machine (Omron HEM-907XL, Omron Healthcare, Lake Forest, IL). Two BP measurements (reported as an average systolic and diastolic value) were taken at 1-min intervals after the participant was seated for 5 min. Self-reported dietary behaviors were measured with items from two validated brief food frequency surveys (10 total items) measuring dietary fat quality [26] and estimated intake of fruits and vegetables [27]. A single item (adapted from the 2 items used in BRFSS) [28] was used to assess usual daily consumption of sugar-sweetened beverage consumption. We gathered self-reported data on physical activity behaviors with a validated adaptation of the RESIDE survey which focuses on walking [29, 30].

### Statistical analysis

For this pilot feasibility study, baseline sample characteristics were summarized using descriptive statistics such as means, percentages, standard deviations etc. Analyses of primary and secondary outcomes and pre-post- changes at 4 months were conducted using paired t-tests. Additionally, we assessed for group differences between males and females in pre-post- changes. To determine our rate of attrition and loss to follow-up, we included in our denominator all enrolled participants who completed the first counseling visit. Since intervention effectiveness outcomes were secondary aims in this study, we did not use any imputation methods to account for missing values but provide a description of those lost to follow-up. For weight and blood pressure measurements taken by CHWs at counseling visits, we used values from the last completed visit when the fourth visit values were missing. For 10 participants, weight and blood pressure values from their last visit after baseline served as their post-intervention values. All analyses were conducted using SAS Version 9.4 (SAS Institute, Cary, NC).

## Results

**Fig. 1:**
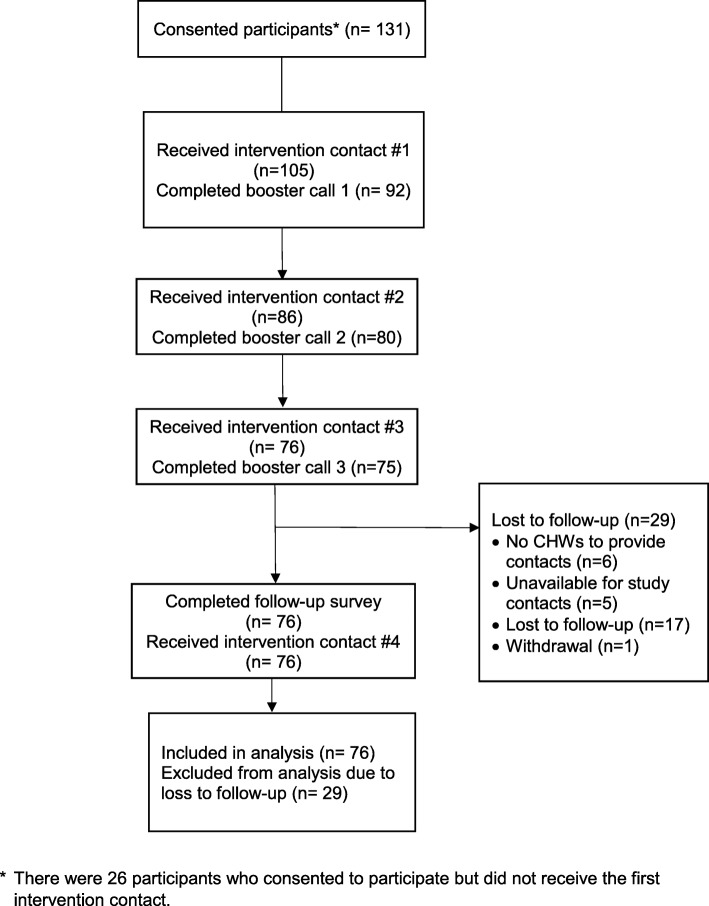
Participant Flow Diagram

Figure 1 shows the flow of participants through the CHANGE intervention. Although 131 participants consented to be in the study, only 105 (80%) completed the first intervention visit, which typically happened on the same day that informed consent was obtained and the baseline survey administered. Among the 105 participants who completed the first counseling visit, 82% completed the second visit, and 72% completed the third and fourth visits. The 29 participants lost to follow-up included 59% for whom we could not ascertain the reason for missing their follow-up visit, 6 participants (21%) who were affected by turnover of the CHWs and the subsequent delay in hiring new staff, 17% who could not be scheduled, and 1 withdrawal from the study (3%). The loss to follow-up was much higher among participants enrolled at the health center compared to the health department (22/62 (35%) vs. 5/43 (12%). Overall, those lost to follow-up included 93% African American women. Program completers did not differ from those lost to follow-up in age, gender or, education. Non-completers, however, included a significantly larger proportion of participants with diagnosed diabetes and taking blood pressure medications (*p* < .01).

Study participants included 62 (59%) from the community health center and 43 (41%) from the local health department. Participant characteristics presented in Table 2 show most participants were Non-Hispanic Black (88%) and female (82%), with a mean age of 62 years. Over half reported having a high school diploma or less in educational attainment. In risk factors for CVD, 79% were diagnosed with hypertension, 32% with diabetes, 56% had hypercholesterolemia, and about 10% were current smokers. The mean blood pressure values were 137 mmHg systolic, and 82 mmHg diastolic; mean weight was 216 lbs. Self-reported physical activity was 80 min per week and dietary behaviors included 3.7 daily servings of fruits and vegetables, 1.8 servings of nuts weekly, and 1.2 (12 oz) servings daily of sugar-sweetened beverages.

**Table 2:** Participant Characteristics

Characteristic (*N* = 105)	N (%) or Mean (SD)
Race/ Ethnicity, %	
Non-Hispanic Black	92 (87.6)
Non-Hispanic White	12 (11.4)
Female, %	86 (81.9)
Age, y	61.9 (10.5)
Education, %	
High school diploma or less	56 (53.3)
Some college	26 (24.8)
College degree (2-year or higher)	23 (21.9)
Physiologic and Behavioral Characteristic, N (%) or Mean (SD)	
Current Smoker, %	11 (10.5)
Diagnosed hypercholesterolemia, %	59 (56.2)
Diagnosed hypertension, %	83 (79.0)
Systolic blood pressure, mm Hg (*n* = 94)	136.6 (22.8)
Diastolic blood pressure, mm Hg (*n* = 94)	81.7 (12.2)
Weight, lb. (*n* = 93)	215.7 (60.7)
Physical activity, minutes/week (*n* = 103)	80.0 (122.9)
Fruit & vegetable servings / d	3.7 (1.7)
Healthy fats, nuts servings / week	1.8 (0.8)
Sugar sweetened beverages / d	1.2 (0.8)

The primary focus of the CHANGE study was the effective *implementation* of an adapted evidence-based intervention. Table 3 includes selected implementation variables related to intervention reach and delivery fidelity. We employed many strategies to recruit patients and community members to the CHANGE program but did not begin collecting data on how participants heard about the study until the last 6 months of implementation. Our recruitment efforts yielded 346 persons who were interested and eligible and of these 131 (38%) enrolled in the program.

**Table 3:** Implementation Effectiveness Outcomes

Implementation Variables	Mean (SD) or N (%)^a^
Recruitment yield (reach) (Enrolled/Interested) (%)	131/346 (38)
Program Delivery	
Contact duration in minutes, mean (SD)	
Counseling visit	76(24)
Booster call	15(14)
Module selected as priority topic for first counseling visit, %	
Medication Adherence	8 (8)
Smoking Cessation	4 (4)
Nutrition (Healthy Eating)	76 (72)
Physical Activity	16 (15)
None selected	1 (1)
Mean modules completed / visit	1.6
Mean goals set / visit	2.2
Mean referrals /visit	0.7
Referral frequency by type	
Wellness center	42 (18)
Senior or Community center	41 (17)
Cooperative Extension programs	16 (7)
Medication assistance	10 (4)
Smoking cessation	7 (3)
Diabetes / other support groups	14 (6)
Farmers market / food resources	38 (16)
Parks / Walking or bike trail	26 (11)
Gym / Walking group	7 (3)
Other community resources	37 (16)
Referrals acted on / referrals given, mean	0.5
Referrals resulting in services received / referrals given, mean	0.4

^a^Percentages may not add to 100 because of rounding

Implementation of the CHANGE program, as measured by delivery fidelity, showed the average counseling visit lasted 76 min and booster calls on average lasted 15 min. Participants (*n* = 105) received 80% (590/735) of the planned visits and phone contacts (see Fig. 1). The proportion of planned contacts completed by each site differed slightly, with the health department participants completing 86% of all planned visits, while the health center participants completed 74%. Overall, 82% (343/420) of home visits and 78% (247/315) of booster calls were completed.

Participants could select the topic (module) considered their top priority and most participants (72%) chose the “Healthy Eating” module, followed by Physical Activity (15%) and Medication Adherence (8%) modules. Each topic module included 1 to 4 sessions (sub-topics) and participants completed on average 1.6 sessions, set 2.2 goals, and received 0.7 referrals per visit. Referrals were made to a variety of community resources, with programs and activities at community-based centers and Cooperative Extension accounting for the largest proportion of referrals (42% combined). Participants given referrals attempted to follow-up on half of those referrals and successfully accessed services for 40% of referrals.

Table 4 shows our effectiveness outcomes (mean changes between pre- and post-intervention measurement) for program completers. We conducted analyses to determine if there were any differences between men and women for each outcome and none were significant. For physiological outcomes, we observed significant mean reductions in both weight and blood pressure. Moreover, among participants with uncontrolled hypertension at baseline, at follow-up 24% (*p* < .001) had systolic pressure < 140 mmHg, and 11% (*p* = .05) reduced their diastolic pressure to < 90 mmHg.

**Table 4:** Program Effectiveness Outcomes

4-Month Outcomes	N^a^	Pre-Intervention Mean (SD)	Post-Intervention Mean (SD)	Mean Change (SD)	*P*-value
Weight, lb.	73	211.8 (59.1)	208.1 (57.5)	−3.8 (13.1)	0.02
Systolic blood pressure (SBP)	76	135.2 (21.7)	129.8 (18.5)	−5.4 (16.5)	0.006
Diastolic blood pressure (DBP)	76	82.5 (12.1)	80.2 (10.4)	−2.3 (9.6)	0.04
Proportion at goal	66^b^				
SBP (< 140), %		57.6	81.8	24.2	0.0002
DBP (< 90), %		72.7	83.3	10.6	0.05
Nuts, healthy fats, servings weekly	76	1.9 (0.8)	2.3 (0.8)	0.4 (1.0)	0.0005
Fruit & vegetable servings, daily	76	4.0 (1.6)	4.9 (1.6)	0.9 (1.70)	< 0.0001
Sugar-sweetened beverages, servings daily	76	1.1 (0.8)	0.6 (0.7)	−0.5 (0.8)	< 0.0001
Physical activity, weekly minutes	71	68.4 (107.9)	108.9 (146)	40.4 (113.9)	0.004

^a^Last visit values used as post-intervention values for participants (*n* = 10) missing weight and blood pressure measures from the last intervention visit

^b^Excludes participants with missing blood pressure values at the last intervention visit

Self-reported dietary and physical activity behaviors also improved significantly. On average, weekly servings of nuts increased by 0.4 servings, and fruits and vegetables by 0.9. Participants also reported lowering their intake of sugar-sweetened beverages by about half a serving daily. For physical activity, participants reported a mean increase of 40 min weekly.

## Discussion

The feasibility phase of the CHANGE study was designed to refine strategies for implementing a CHW-delivered, evidence-based, CVD intervention that also strengthens clinical-community linkages. Our findings from this phase not only demonstrate effectiveness in both implementation and intervention outcomes, but also identify opportunities to improve implementation strategies.

Implementation effectiveness was our primary focus with the purpose of refining our plans for implementation with a larger study sample. From this pilot we gathered information to guide our recruitment and retention of participants as well as identify processes for linking participants to community resources that would support CVD risk reduction efforts (i.e., clinical-community linkages). With our focus on recruiting from both community and clinical settings, we enrolled 38% of adults eligible and willing to be contacted about participation. In a similar study of CVD risk reduction in both public health and healthcare settings, [18] where CHWs provided point-of-service screening, education, and care coordination to mostly rural White and Hispanic residents, 27% of those with complete screening records had an initial screening visit over a one-year period. Among those screened and identified as being at risk for CVD, 53.5% received a medical or lifestyle referral [18]. While meaningful differences between this study and ours limit direct comparisons, our reach in this feasibility phase suggests the recruitment strategies used were reasonable, but could be improved. Our recruitment of clinical patients was hampered by not having the CHW well-integrated as a valued member of the agency’s health care delivery team, which made timely recruitment and enrollment difficult. A possible strategy used by Krantz and colleagues to improve clinic patient referrals, would be to identifying a ‘physician champion.’ [18]

A key component of the CHANGE intervention was linking participants to community resources that would support CVD risk reduction behaviors and following up with participants about referrals given. Making these community-clinical linkages is particularly important in communities like Hertford that are medically underserved and negatively impacted by many social factors that drive health. Although there is evidence that strengthening these linkages leads to improved health outcomes achieved through changes in lifestyle behaviors, [20] there is still much to be learned about how best to operationalize these linkages in rural settings where accessing services is challenging. Moreover, additional research is needed to identify effective models of leveraging the role of CHWs in community-clinical linkages and enhancing CHW training to expand the field [20].

Another key goal of this feasibility study was to assess the implementation effectiveness of a CHW-delivered intervention in participant retention and receipt of the planned intervention dose. While participants received 80% of planned intervention contacts, our retention rate of 72% (28% lost to follow-up) was suboptimal, though not unusual in similar studies conducted in rural settings. For example, in a study by Seguin and colleagues among rural women (95% white) receiving a CVD prevention program delivered by community members, the mean attendance was 74%, with 22% of participants lost to follow-up [20]. In the Krantz study [18], only 15% of screened participants returned for retesting. Moreover, this study also found site differences (public health vs. healthcare agency) in program uptake, with lower program uptake observed in clinic participants compared to those from health departments [18]. We observed similar site differences in our loss to follow-up rate, with clinic participants having a higher rate of 35% compared to 12% among health department participants. While we suspect that being recruited by a CHW who is a trusted community member, vs. a referral by clinical staff may explain some of these differences, there are likely other explanatory factors. One potential strategy for recruiting and retaining clinic participants may be to have the CHW play a bigger role in participant engagement earlier in the referral process.

Effectiveness in program outcomes was a secondary aim of this study because of the adaptions made. With CHWs delivering an adapted evidence-based intervention we observed significant mean changes in our targeted CVD risk factors. Moreover, our mean reductions in blood pressure and weight were similar to those observed in the Heart-to-Health in-person counseling arm, [23] and the evidence-based intervention on which CHANGE is based. Compared to CHW interventions included in a recent systematic review [15] our findings are also encouraging for blood pressure outcomes. In this review, among studies with higher quality designs, the median decrease in systolic blood pressure was 6.0 to 2.2 mmHg depending on the presence or absence of a team-based approach to patient clinical care; for diastolic pressure the median changes were 1.1 to 1.3 mmHg [15]. In CHANGE where the CHWs did not work alongside physicians and nurses (e.g., team-based care), our median decrease in systolic and diastolic blood pressure was 3.5 and 3.0 mmHg, respectively.

Our preliminary effectiveness findings can also be compared to CVD risk reduction intervention studies conducted in rural settings, although not among Non-Hispanic Blacks. In the Heart of New Ulm Project (HONU), [17] a community-based CVD prevention project, they observed improvement is blood pressure, lipids, and 10-year ASCVD risk score in program participants compared to controls. Mean systolic blood pressure was changed by − 0.7 mmHg, and diastolic by − 1.7 mmHg, [17] compared to a change of − 5.4 and − 2.3 mmHg, respectively in CHANGE. Similarly, Krantz and colleagues found a − 1.1 kg (2.6 lb.) change in weight, − 3.8 mmHg change in systolic and − 2.3 in diastolic pressure, which are all comparable our findings. For 10-year Atherosclerotic Vascular Disease (ASCVD) risk score, the study by Seguin and colleagues in rural populations (among mostly non-Hispanic whites) showed lowering of risk scores by − 0.96 [19]. Although we do not report ASCVD risk scores (because of the small clinical sample size), the blood pressure lowering observed in CHANGE would result in a relative risk reduction of 0.73 to a 10-year ASCVD risk score [31]. While our 3.8-lb. weight loss would have only a minimal effect on CVD risk reduction, the increased consumption of vegetables and nuts could reduce risk further by as much as 30% [32]. Through our data sharing agreement in this feasibility phase, we were able to calculate the risk scores for the clinical sample and refined our strategies for gathering the data from the electronic health record system to be used in our next phase of implementation.

The findings of this feasibility study cannot be fully interpreted without mentioning a few noteworthy limitations. First, our use of a single-arm, pre-post study design means we cannot distinguish between the intervention being responsible for the effect observed versus alternate explanations (e.g., a placebo effect or contributions from other community-level factors). Given that this intervention was already found to be effective in a comparative effectiveness study, we elected to focus this study on feasibility with the goal of obtaining preliminary evidence of effectiveness of the intervention when adapted for delivery by CHWs in a rural context. It should be noted that even though CHWs have been recommended for delivery of interventions to prevent CVD, the Community Preventive Services Task Force [15] identified a gap in the evidence for “interventions conducted in rural areas” and knowledge of “whether CHWs are effective in helping patients access care for their CVD risk factors, especially patients from medically underserved groups.” This study helps to fill these evidence gaps and does so with a sample mostly rural non-Hispanic Blacks. Second, we observed a high level of attrition in the clinic sample and identified that those loss to follow-up were more likely to have diagnosed diabetes and hypertension. While there is potential for this attrition to bias the study results, we suspect that reasons for participants not completing the final program visit are likely unrelated to the program itself, given its delivery by home visits and the low intensity. Third, there is the potential for regression to the mean in our blood pressure findings due to the priority selection of clinic patients with uncontrolled hypertension. Though we report paired t-test results for blood pressure changes, we conducted additional analyses using regression analysis to account for pre-test values (i.e., adjusting for baseline value) and found that our findings did not change. Despite these limitations, this feasibility study fulfilled our aims of identifying key refinements needed for more effective implementation of the CHANGE program in a larger study sample.

## Conclusions

In summary, initial evidence for the implementation and program effectiveness of CHANGE provide preliminary support for CHW-delivery of the intervention to reduce CVD risk factors among a rural, predominantly African American population. If results from this feasibility study can be enhanced in a larger sample, there would be significant potential to positively impact the excess burden of chronic disease that adversely impacts rural, low-income populations. Implications for refinements in the follow-up phase include: 1) pre-implementation planning for staff turnover at partnering health agencies; 2) improved training of CHWs and their supervisors; and 3) focus on integrating CHWs into the health care delivery team.

## Data Availability

The deidentified datasets analyzed in the study reported are available from the corresponding author on reasonable request.

## Abbreviations

BP: Blood Pressure
CHANGE: Carolina Heart Alliance Networking for Greater Equity
CHW: Community Health Worker
CVD: Cardiovascular disease
EHR: Electronic Health Record
FQHC: Federally Qualified Health Center
HIPAA: Health Insurance Portability and Accountability Act
NC: North Carolina
UNC: University of North Carolina

## References

[1] The US (2018). Burden of disease collaborators, Mokdad AH, Ballestros K, et al. the state of US health, 1990-2016: burden of diseases, injuries, and risk factors among US states. JAMA..

[2] Centers for Disease Control and Prevention (CDC). Interactive Atlas of Heart Disease and Stroke. https://nccd.cdc.gov/DHDSPAtlas/. Accessed 13 Apr 2019.

[3] Dwyer-Lindgren L, Stubbs RW, Bertozzi-Villa A (2017). Inequalities in life expectancy among US counties, 1980 to 2014: temporal trends and key drivers. JAMA Intern Med.

[4] Howard G, Labarthe DR, Hu J (2007). Regional differences in African Americans' high risk for stroke: the remarkable burden of stroke for southern African Americans. Ann Epidemiol.

[5] Dubay LC, Lebrun LA (2012). Health, behavior, and health care disparities: disentangling the effects of income and race in the United States. Int J Health Serv.

[6] Meit M, Knudson A, Gilbert T, et al. The 2014 Update of the rural-urban chartbook. Rural Health Reform Policy Research Center. https://ruralhealth.und.edu/projects/health-reform-policy-research-center/pdf/2014-rural-urban-chartbook-update.pdf. Accessed 13 Apr 2019.

[7] Lee-Kwan SH, Moore LV, Blanck HM (2017). Disparities in state specific adult fruit and vegetable consumption - United States, 2015. MMWR Morbidity and Mortal Weekly Report.

[8] Li Yanping, Pan An, Wang Dong D., Liu Xiaoran, Dhana Klodian, Franco Oscar H., Kaptoge Stephen, Di Angelantonio Emanuele, Stampfer Meir, Willett Walter C., Hu Frank B. (2018). Impact of Healthy Lifestyle Factors on Life Expectancies in the US Population. Circulation.

[9] Dai S, Carroll DD, Watson KB (2015). Participation in types of physical activities among US adults--National Health and nutrition examination survey 1999-2006. J Phys Act Health.

[10] Pilkerton CS, Singh SS, Bias TK, Frisbee SJ (2017). Healthcare resource availability and cardiovascular health in the USA. BMJ Open.

[11] Rogers CK, Zhang NJ (2017). An early look at the association between state Medicaid expansion and disparities in cardiovascular diseases: a comprehensive population health management approach. Popul Health Manag.

[12] Knopf TNC (2018). Rural health by the numbers. North Carolina Health News.

[13] Smith SA, Ansa B (2016). A systematic review of lifestyle interventions for chronic diseases in rural communities. J Ga Public Health Assoc.

[14] Cai Y, Richards EA (2016). Systematic review of physical activity outcomes of rural lifestyle interventions. West J Nurs Res.

[15] Community Preventive Services Task Force (2015). Cardiovascular Disease Prevention and Control: Interventions Engaging Community Health Workers.

[16] American Public Health Association. Community Health Workers. https://www.apha.org/apha-communities/member-sections/community-health-workers. Accessed 13 Apr 2019. 10.2190/NS.20.3.l 20943481

[17] Sidebottom AC, Sillah A, Miedema MD (2016). Changes in cardiovascular risk factors after 5 years of implementation of a population-based program to reduce cardiovascular disease: the heart of New Ulm project. Am Heart J.

[18] Krantz M, Coronel SM, Whitley EM (2013). Effectiveness of a community health worker cardiovascular risk reduction program in public health and health care settings. Am J Public Health.

[19] Seguin RA, Paul L, Folta SC (2018). Strong hearts, healthy communities: a community-based randomized trial for rural women. Obesity..

[20] Lohr AM, Ingram M, Nuñez AV (2018). Community–clinical linkages with community health workers in the United States: a scoping review. Health Promot Pract.

[21] Curran GM, Bauer M, Mittman B (2012). Effectiveness-implementation hybrid designs: combining elements of clinical effectiveness and implementation research to enhance public health impact. Med Care.

[22] Thiese MS (2014). Observational and interventional study design types; an overview. Biochem Med.

[23] Keyserling TC, Sheridan SL, Draeger LB (2014). A comparison of live counseling with a web-based lifestyle and medication intervention to reduce coronary heart disease risk: a randomized clinical trial. JAMA Intern Med.

[24] United States Census Bureau (2018). QuickFacts: Hertford County, North Carolina.

[25] Robert Wood Johnson Foundation Program (2019). County Health Rankings & Roadmaps: Hertford.

[26] Kraschnewski JL, Gold AD, Gizlice Z (2013). Development and evaluation of a brief questionnaire to assess dietary fat quality in low-income overweight women in the southern United States. J Nutr Educ Behav.

[27] Block G, Gillespie C, Rosenbaum E, Jenson C (2000). A rapid food screener to assess fat and fruit and vegetable intake. Am J Prev Med.

[28] Centers for Disease Control and Prevention (2017). Behavioral Risk Factor Surveillance System Questionnaire.

[29] Giles-Corti B, Timperio A, Cutt H (2006). Development of a reliable measure of walking within and outside the local neighborhood: Reside’s neighborhood physical activity questionnaire. Prev Med.

[30] Jones S, Evenson K, Johnston L (2014). Psychometric properties of the modified RESIDE physical activity questionnaire among low-income overweight women. J Sci Med Sport.

[31] Lloyd-Jones DM, Huffman MD, Karmali KN (2017). Estimating longitudinal risks and benefits from cardiovascular preventive therapies among Medicare patients the Million Hearts longitudinal ASCVD risk assessment tool: A special report from the American Heart Association and American College of Cardiology. J Am Coll Cardiol.

[32] Estruch R, Ros E, Salas-Salvado J (2013). Primary prevention of cardiovascular disease with a Mediterranean diet. N Engl J Med.

